# Resectable and Borderline Resectable Pancreatic Ductal Adenocarcinoma: Role of the Radiologist and Oncologist in the Era of Precision Medicine

**DOI:** 10.3390/diagnostics11112166

**Published:** 2021-11-22

**Authors:** Federica Vernuccio, Carlo Messina, Valeria Merz, Roberto Cannella, Massimo Midiri

**Affiliations:** 1 Radiology Unit, University Hospital “Paolo Giaccone”, 90127 Palermo, Italy; 2 Oncology Unit, A.R.N.A.S. Civico, 90127 Palermo, Italy; carlo.messina@arnascivico.it; 3 Department of Medical Oncology, Santa Chiara Hospital, 38122 Trento, Italy; valeriamerz@gmail.com; 4 Department of Biomedicine, Neuroscience and Advanced Diagnostics (BIND), University Hospital of Palermo, Via del Vespro 129, 90127 Palermo, Italy; rob.cannella89@gmail.com (R.C.); massimo.midiri@unipa.it (M.M.); 5 Department of Health Promotion, Mother and Child Care, Internal Medicine and Medical Specialties (PROMISE), University of Palermo, Via del Vespro 129, 90127 Palermo, Italy

**Keywords:** pancreatic ductal adenocarcinoma, pancreatic neoplasm, computed tomography (CT), magnetic resonance imaging (MRI)

## Abstract

The incidence and mortality of pancreatic ductal adenocarcinoma are growing over time. The management of patients with pancreatic ductal adenocarcinoma involves a multidisciplinary team, ideally involving experts from surgery, diagnostic imaging, interventional endoscopy, medical oncology, radiation oncology, pathology, geriatric medicine, and palliative care. An adequate staging of pancreatic ductal adenocarcinoma and re-assessment of the tumor after neoadjuvant therapy allows the multidisciplinary team to choose the most appropriate treatment for the patient. This review article discusses advancement in the molecular basis of pancreatic ductal adenocarcinoma, diagnostic tools available for staging and tumor response assessment, and management of resectable or borderline resectable pancreatic cancer.

## 1. Introduction

The American Cancer Society and the European Society for Medical Oncology estimate that in 2021 about 48,220 people in the US and 42,300 in Europe will die of pancreatic ductal adenocarcinoma (PDAC) [1,2]. The incidence and mortality are growing over time, with a 5-year relative survival of 10.8%, and it is estimated that PDAC will become the second leading cause of cancer deaths in the US in the next 20–30 years [3]. In this scenario, many efforts have been made to understand the genetics of precancerous lesions, for identification and follow-up of precancerous lesions, for early detection of PDAC, as well as for identifying new patient-tailored chemotherapy regimens for PDAC based on genetic mutations of the tumor itself [3,4,5].

**Figure 1:**
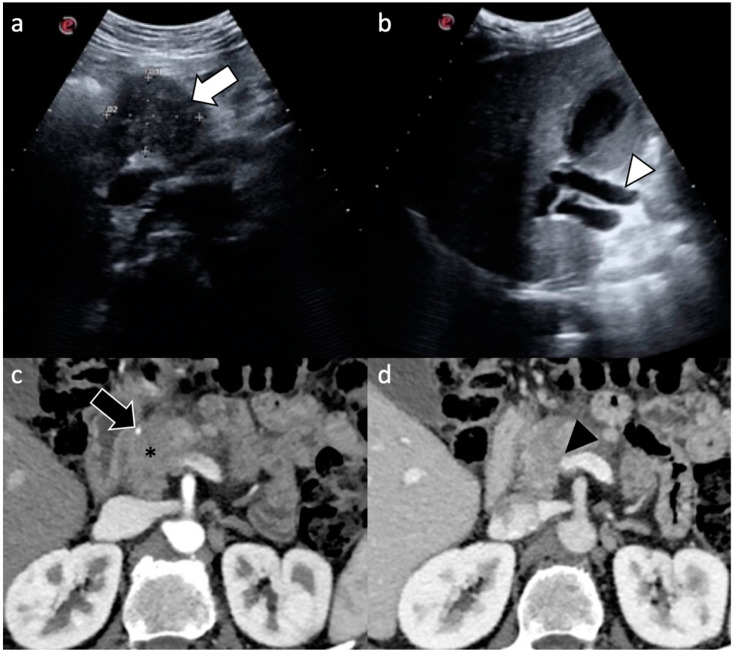
A 57-year-old man who came to the emergency department for jaundice and abdominal pain. (**a**,**b**) US detected a mass in the pancreatic head (white arrow) causing upstream dilatation of the common bile duct (white arrowhead); (**c**,**d**) Pancreatic CT scan confirmed the presence of mass in the pancreatic head (*) that caused encasement of the gastroduodenal artery (black arrow) as well as encasement and narrowing of the superior mesenteric-portal venous confluence (black arrowhead), the superior mesenteric vein, and the portal vein.

The management of patients with PDAC involves a multidisciplinary team, ideally including experts from surgery, diagnostic imaging, interventional endoscopy, medical oncology, radiation oncology, pathology, geriatric medicine, and palliative care [6]. Radiologists play a pivotal role in the decisions taken by the multidisciplinary team during the different steps of patient management (i.e., diagnosis, staging, and therapeutic monitoring) and their role has evolved in parallel with advances in clinical management [6,7,8]. An adequate staging of PDAC and re-assessment of the tumor after neoadjuvant therapy allows the multidisciplinary team to choose the most appropriate treatment for the patient [6]. Although PDAC may be detected at ultrasound (US), contrast-enhanced computed tomography (CT) is the recommended imaging technique for dedicated pancreatic imaging for diagnosis, staging, and follow-up (Figure 1) [6]. About 30% of patients with localized, non-metastatic PDAC may have indeterminate liver lesions at the time of diagnosis and may require further investigations [9]. Dual-energy CT or magnetic resonance imaging (MRI) may be helpful for characterization of indeterminate liver lesions [6,10].

State-of-the-art knowledge of the advances in molecular basis of PDAC; the diagnostic tools available for staging and tumor response assessment with potential imaging pitfalls; and management of resectable or borderline resectable PDAC are highly relevant for the radiologists. This review article discusses these aspects with the aim of enhancing our value as radiologists in the clinical management of resectable and borderline resectable PDAC.

## 2. Assessment of Resectability and Implications for Patient Management

### 2.1. Role of Radiologist

The imaging presentation of PDAC is summarized in Table 1 [6,11]. CT and MRI have a sensitivity of about 67–100% for the detection of PDAC > 2 cm; however, the sensitivity drops to 50–78% in the case of smaller tumors [12,13]. In particular, it is well known that PDAC detection is limited in case of small and isoattenuating noncontour-altering tumors, which comprise nearly 30% of lesions smaller than 2 cm [14]. Pathologic findings of isoattenuating PDAC differ from those of usual PDAC, due to lower tumor cellularity, more frequent intratumoral acinar tissue and islet cells, and less prominent tumor necrosis [14].

Dual-energy CT proves to be helpful in increasing conspicuity of hypovascular PDAC with low kVp techniques and low energy virtual monochromatic images, as well as increasing the accuracy of tumor measurements [15,16,17]. In addition, the adoption of iodine maps allows to quantify iodine uptake of PDAC, and this quantification seems superior to CT attenuation measurements in the assessment of tumor response [18]. In addition to the benefit for tumor assessment, the adoption of dual-energy CT may be helpful for patient-tailored protocol optimization. Specifically, given that low energy virtual monocromatic images have high contrast-noise ratio, it is possible to improve image contrast when the intravenous contrast bolus is suboptimal, such as in case of reduced iodinated contrast bolus in the setting of renal insufficiency or when the bolus timing is not accurate [19]. In addition, it has been proven by pilot results that the diagnostic performance for PDAC detection of a simulated twin-phase pancreatic protocol CT generated from a single portal venous phase dual-energy CT is comparable to the standard two-phase protocol (i.e., pancreatic phase and portal venous phase), thus allowing for a significant reduction in radiation dose [20].

CT perfusion (CTP) consists of the dynamic acquisition after injection of a contrast agent, enabling quantification of tissue vascularization. CTP could improve the diagnostic workup of PDAC by combining functional information and spatial detail. As demonstrated by a recent systematic review [21], CTP can accurately distinguish PDAC from non-tumorous pancreatic parenchyma, since PDAC has significantly lower BF and BV compared to normal pancreatic parenchyma. CTP parameters seem to improve the detection of isoattenuating PDAC, and might be helpful as a biomarker for the pathological grade [21].

More recently, a huge effort is also being made to adopt deep learning for improving detection of PDAC [22]. Liu et al. [22] reported a very high accuracy of a convolutional neural network for the diagnosis of PDAC, yielding a sensitivity of 99% and specificity of 98.9% in the local test set, and a sensitivity of 79% and specificity of 97.6% in a cross-racial external validation set. Specifically, the sensitivity of the convolutional neural network for tumors smaller than 2 cm was 92.1% in the local test sets and 63.1% in the external validation test set. However, in this study, the study cohort included only patients with PDAC and patients with a normal pancreas, thus introducing a selection bias from the exclusion of other pancreatic diseases that may pose diagnostic challenges in clinical practice. Indeed, PDAC needs to be differentiated from many benign pancreatic/peripancreatic lesions and anatomic variants, such as pancreatitis, intrapancreatic splenosis, focal fat, and from other malignancies, such as metastases [8,23,24,25].

Surgical and radiologic criteria for resectability are currently based on anatomic criteria alone. Anatomical definition of borderline resectable PDAC is a tumor that is at high risk for margin-positive resection (R1, R2) when surgery is used as an initial treatment strategy [26]. The adoption of anatomical criteria for resectability has a significant role in the prediction of overall survival [27]. Based on National Comprehensive Cancer Network^®^ (NCCN) guidelines, all patients with a diagnosis of PDAC should undergo contrast-enhanced CT for tumor staging and assessment of resectability within 4 weeks of surgery and following neoadjuvant treatments [6]. A CT structured reporting template is nowadays recommended by many international societies [6,28], as it allows the reduction in the number of missing morphological and vascular features, and the improvement of inter-reader agreement compared to free-text reports [29]. Recently, a deep learning image reconstruction algorithm has been developed for CT assignment of the local resectability of PDAC with good results [30].

**Figure 2:**
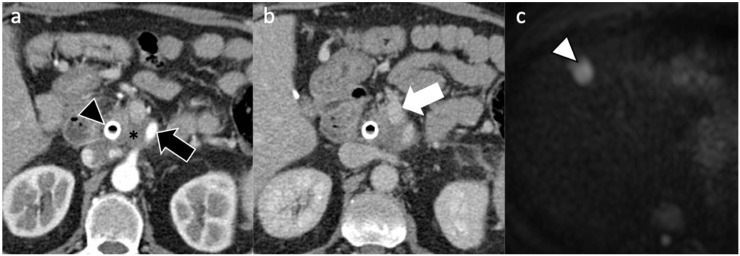
A 61-year-old man with non-resectable PDAC. Pancreatic CT scan on (**a**) arterial and (**b**) portal venous phases shows the presence of a biliary stent (black arrowhead) and a pancreatic mass (*) causing encasement of both superior mesenteric artery (black arrow) and vein (white arrow). The patient commenced modified FOLFIRINOX regimen at diagnosis. However, after 6 months, (**c**) liver MRI on diffusion weighted imaging showed appearance of liver metastasis in segment IV (arrowhead in c).

A CT structured reporting template for PDAC should include assessment of the following items (Figure 2) [28]:

‑ Morphologic evaluation (size, appearance, location, pancreatic duct narrowing/abrupt cutoff with or without upstream dilatation, biliary tree abrupt cutoff with or without upstream dilatation, gallbladder dilatation);
‑ Arterial evaluation, including contact with celiac axis, common hepatic artery, gastroduodenal artery, splenic artery, superior mesenteric artery, or arterial variants;
‑ Venous evaluation, including contact with main portal vein, superior mesenteric vein, splenic vein, and inferior cava vein, and other factors (thrombus within vein, venous collaterals);
‑ Extrapancreatic evaluation, including liver lesions, peritoneal or omental nodules, ascites, suspicious lymph nodes, and invasion of adjacent structures;
‑ Final impression of local tumor resectability, vascular contact, and presence of metastasis.

In regard to liver metastases, preoperative MRI, especially diffusion weighted imaging, has been found to depict synchronous small liver metastases that are undetectable with standard workup CT in approximately 10–24% of patients; this improvement in the detection of liver metastases may change patient management with decrease in the rate of unnecessary laparotomy and pancreatectomy [31,32,33].

### 2.2. Resectable Pancreatic Cancer

Resectable PDAC does not show any arterial or venous tumor contact, or may show a venous contact below 180° without vein contour irregularity, but with a lack of any arterial contact [6]. Resectable PDACs are usually candidates for surgery as first approach. However, the decision is always discussed at multidisciplinary meetings because other factors are considered, including clinical and radiological features [34]. Indeed, the decision about the appropriateness of resection depends on patient ability to withstand the physiological challenges of surgery with a clinical assessment that includes patient performance status and comorbidities [26,35]. From a radiological standpoint, about 37% of patients with a PDAC deemed resectable on CT will turn out to have margin-positive resection at surgery [36]. This may be partially related to the considerable inter-observer variability in the assessment of resectability at CT, even among experienced radiologists [37], but also other tumor factors play a role. Indeed, larger tumor size (>4 cm) and tumor abutment to the portomesenteric vein are associated with margin-positive resection in these resectable patients [36]. Interestingly, Kim et al. [38] developed and validated an easy risk score including five variables for estimating recurrence and predicting prognosis at 1 year in patients with resectable PDAC who undergo upfront surgery. This risk score is based on CT features and includes tumor size (cutoffs of 2 cm and 4 cm), tumor density on portal venous phase (hypodense or iso/hyperdense), tumor necrosis, peripancreatic tumor infiltration, and suspicious metastatic lymph nodes [38]. The validity of the score is likely due to the correlation between CT features, pathologic findings, and prognosis in PDAC [38]:

‑ Tumor hypodensity is associated with poorer tumor differentiation, tumor necrosis with poorer tumor differentiation, lymph node metastasis, and lymphovascular invasion;
‑ Suspicious metastatic lymph nodes on CT with lymph node metastases at pathology, lymphovascular invasion, and perineural invasion;
‑ Peripancreatic tumor infiltration with positive pathologic resection, lymphovascular invasion, and perineural invasion.

Radiologists need to be aware of the clinical relevance of these CT features and should carefully report them to the surgeon and the oncologist in the multidisciplinary team. Indeed, it is necessary to adequately stage the disease to provide optimal cancer care and prognostication.

### 2.3. Borderline Resectable Pancreatic Cancer

The definition of borderline resectable tumor is still controversial and varies among societies, radiologists, and surgeons [6,26,39,40,41,42]. It is generally agreed that borderline resectable PDACs are neither clearly resectable nor clearly unresectable, but may benefit from neoadjuvant therapy and are more likely to require a vascular resection at the time of pancreatoduodenectomy. Herein, we report the definition of borderline resectable PDAC based on NCCN guidelines version 2.2021 because NCCN guidelines are reviewed and updated on a continuing basis to ensure compliance with the most current evidence, and they provide comprehensive recommendations from head to toe with a multidisciplinary approach [6]. Neoadjuvant chemotherapy and/or radiotherapy is considered to increase the chances of an R0 resection and is usually considered in patients with borderline resectable PDAC [6].

The definition of borderline resectable PDAC needs careful evaluation of arterial and venous involvement. The assessment of arterial involvement is divided based on the location of the tumor (pancreatic head/uncinate or pancreatic body/tail) and the solid tumor contact with vessels (i.e., tumor contact or increased hazy density/stranding of the fat surrounding peripancreatic vessels). In patients with tumors in the pancreatic head or uncinate process, borderline resectable disease is defined as (1) solid tumor contact with the common hepatic artery without extension to the celiac axis or hepatic artery bifurcation, (2) ≤180° involvement of the superior mesenteric artery and/or celiac axis, and (3) solid tumor contact with variant arterial anatomy. In patients with tumors in the pancreatic body or tail, borderline resectable disease is defined as (1) solid tumor contact with the celiac axis ≤180° or (2) involvement of the celiac axis greater than 180° of the aorta and gastroduodenal artery are uninvolved, and the surgeons are able to perform an arterial anastomosis (modified Appleby procedure with resection of the mass and the celiac axis en bloc) [6,43]. This latter criterion for the definition of resectability is still debated, as this procedure is performed only in dedicated centers. In the NCCN guidelines, it is specified that some panel members prefer to put the solid tumor contact with the celiac axis >180° in the locally advanced category tout court [6]. Other major societies (e.g., MD Anderson Cancer Center, Alliance for Clinical Trials in Oncology, and American Hepato-Pancreato-Biliary Association/Society for Surgery of the Alimentary Tract/Society for Surgical Oncology) consider a pancreatic tumor unresectable in the case of encasement of the celiac axis [39,40,41,44,45]. The International Association of Pancreatology put this criterion among the unresectability criteria, yet specify that some members would prefer to put this criterion in the borderline resectable category [26]. There are some other differences between NCCN guidelines and the consensus of the International Association of Pancreatology in regard to the definition of borderline resectable PDAC for arterial involvement. Indeed, arterial involvement is defined as borderline resectable in case of (a) tumor contact of less than 180° with superior mesenteric artery or celiac axis without deformity/stenosis, or (b) tumor contact with common hepatic artery without showing tumor contact of the proper hepatic artery and/or celiac artery [26]. Differently from NCCN, the presence of variant arterial anatomy is not taken into consideration [26].

In regard to venous involvement, borderline resectable PDAC is considered in the case of a solid tumor contact with the inferior vena cava or a tumor contact with the superior mesenteric vein or portal vein equal or less than 180°, but with contour irregularity of the vein or thrombosis or a contact with the superior mesenteric vein or portal vein more than 180°, but with suitable vessel proximal and distal to the site of involvement, allowing for safe and complete resection and vein reconstruction [6]. However, the sentence “allowing for safe and complete resection and vein reconstruction” may be ambiguous, and is usually discussed at the multidisciplinary board with a face-to-face discussion between the surgeons and the radiologists. With the aim of clarifying this ambiguous sentence, an international consensus of the International Association of Pancreatology on the classification of borderline resectable PDAC has included the “duodenal margin criteria” for determination of “resectability” of portal vein or superior mesenteric vein invasion as a surrogate to a more refined knowledge of the venous tributaries [18]. Based on this international consensus, the tumor is defined as borderline resectable in the case of abutment or invasion of the portal vein or superior mesenteric vein with bilateral narrowing or occlusion, not exceeding the inferior border of the duodenum, while it is considered unresectable/locally advanced if this venous tumor contact exceeds the inferior border of the duodenum [26]. The NCCN Clinical Practice Guidelines in Oncology Version 2.2017 proposed that borderline resectable PDAC should include a lack of contact with the most proximal draining jejunal branch into the superior mesenteric vein. However, the first and the second jejunal vein usually form a common trunk, and assessment of this criterion on CT posed many challenges, and, therefore, is not included either in the International Association of Pancreatology consensus on definition and criteria of borderline resectable pancreatic ductal adenocarcinoma 2017 nor in the most recent version of NCCN guidelines (i.e., version 2.2021) [6,26].

## 3. Management of Resectable Pancreatic Cancer

Surgery with radical intent represents the only chance of cure for approximately 20% of localized PDAC considered resectable at diagnosis [46]. However, the 5-year survival rate achieved by upfront surgery is only 10% [47].

The current standard therapeutic strategy of resectable disease is upfront surgery, followed by adjuvant chemotherapy in patients with macroscopic complete removal of PDAC [48,49]. The ESPAC-1 trial was the first trial demonstrating a significant survival benefit by using adjuvant fluorouracil in patients with resected pancreatic cancer (five-year survival rate 21 percent compared to 8 percent among patients who did not receive chemotherapy, *p* = 0.009) [50]. Gemcitabine has also been shown to significantly enhance median disease-free survival (DFS) and overall survival (OS) when compared to observation alone (13.4 versus 6.7 months and 22.8 versus 20.2 months, respectively) [47]. The phase 3 ESPAC-3 version2 trial did not report a different survival between adjuvant fluorouracil plus folinic acid compared with gemcitabine (23.0 versus 23.6 months) after resection of pancreatic ductal adenocarcinoma [51]. In the phase 3 ESPAC-4 trial the combination of gemcitabine and capecitabine (GEMCAP) demonstrated longer OS compared with gemcitabine monotherapy after resection of pancreatic adenocarcinoma (28.0 versus 25.5 months, respectively), even if the lack of a postoperative restaging and CA19.9 level limits were the main points of weakness [52]. More recently, the phase 3 multicenter PRODIGE 24/CCTG PA trial compared a modified FOLFIRINOX regimen (oxaliplatin, irinotecan, leucovorin, and fluorouracil) with gemcitabine in patients with pancreatic ductal adenocarcinoma [53]. Inclusion criteria comprised patients with PS ECOG 0 or 1, R0 and R1 resected, with pN0 and pN1 status, with serum CA 19-9 level of 180 U per milliliter or lower.

The benefit of radiotherapy in the adjuvant setting is controversial. The tolerability and benefit of adjuvant chemoradiotherapy in patients receiving adjuvant mFOLFIRINOX are unclear. In the ESPAC-1 trial, patients assigned to chemoradiotherapy had shorter survival outcomes than those who did not receive it [50]. American ASCO and ASTRO guidelines suggest adding postoperative chemoradiotherapy to adjuvant chemotherapy for patients with node-positive or margin-positive disease [54,55].

However, almost half of patients who underwent adjuvant chemotherapy relapsed within two years [45]. The biological explanation of the high recurrence rate is that PDAC should probably be considered a metastatic disease ab initio [56].

Multidisciplinary treatment of resectable pancreatic cancer is moving towards neoadjuvant or perioperative treatment. A propensity score matched analysis carried out on 15.237 patients reported an improved survival using neoadjuvant therapy followed by resection compared to upfront surgery [57]. Meanwhile, two recent meta-analyses investigating the putative role of a neoadjuvant strategy compared to standard upfront resection failed to demonstrate a statistically significant survival benefit [58,59].

The randomized phase 3 PREOPANC trial was the first and largest prospective study to compare a perioperative chemoradiotherapy strategy with gemcitabine to upfront surgery followed by adjuvant gemcitabine in patients with resectable or borderline resectable PDAC [60]. Although the PREOPANC study did not demonstrate a statistically significant longer OS in the neoadjuvant arm, the secondary endpoints, including DFS survival and R0 resection rate, were superior in the experimental arm, suggesting a potential benefit of this approach. A neoadjuvant/perioperative strategy with modified FOLFIRINOX has recently been proven feasible in resectable or borderline resectable PDAC patients in the phase 2/3 NEPAFOX trial [61].

Recently, the randomized phase II SWOG S1505 trial showed similar efficacy with perioperative modified FOLFIRINOX and gemcitabine plus nab-paclitaxel, none of which reached the preplanned 2-year survival rate [62]. In the Italian PACT-15 trial, a perioperative combination regimen (PEXG) provided a higher 1-year event-free survival compared to adjuvant PEXG or gemcitabine [63].

Several ongoing trials are assessing the efficacy of preoperative or perioperative therapy in patients with resectable and borderline resectable PDAC aiming to improve survival outcomes [64,65,66,67,68].

## 4. Management of Borderline Resectable and Locally Advanced Pancreatic Cancer

International guidelines recommend the use of a preoperative treatment in borderline resectable and locally advanced PDAC [48,49,69,70]. Metanalyses including cohort studies, phase 1/2 trials, and retrospective series have shown higher radical resection rates and better survival outcomes using a neoadjuvant approach in borderline resectable PDAC [71,72,73].

In the four arm, phase 2 ESPAC-5F trial, borderline resectable PDAC patients were randomized to upfront surgery, neoadjuvant GEMCAP, FOLFIRINOX, or chemoradiation. No difference in resection rate, i.e., the primary endpoint, was observed. However, the neoadjuvant strategy seemed to prolong survival compared to immediate resection [74].

As forementioned, the PREOPANC trial failed to show a benefit in terms of OS with a perioperative multimodality strategy compared to adjuvant gemcitabine in resectable and borderline resectable PDAC [60]. However, a preplanned subgroup analysis indicated a longer OS after preoperative chemoradiotherapy for the borderline resectable subpopulation.

The feasibility of a preoperative triplet regimen followed by chemoradiation for patients with borderline resectable and locally advanced PDAC patients has been explored in a prospective, multicenter, single-arm trial and in a phase 2 trial [75,76]. Upfront neoadjuvant chemoradiation has been compared with immediate surgery in borderline PDAC, demonstrating increased survival and R0 resection rates [77].

However, the role of neoadjuvant chemoradiation remains controversial. In the phase 2 Alliance A021501 trial, the addition of stereotactic body radiation therapy to neoadjuvant mFOLFIRINOX failed to improve OS [78].

Similarly, in locally advanced tumors, an induction therapy could lead to conversion surgery [79]. The activity of gemcitabine plus nab-paclitaxel in the locally advanced setting has been supported in the phase 2 LAPACT trial, in which after six cycles of induction chemotherapy investigators could choose between continuing chemotherapy or candidate patients for chemoradiation or surgery [80].

The phase 2 NEOLAP trial failed to demonstrate a statistically relevant difference in conversion rate by administering FOLFIRINOX compared to gemcitabine plus nab-paclitaxel after an induction chemotherapy with gemcitabine plus nab-paclitaxel doublet in locally advanced pancreatic cancer [81].

Whether chemoradiotherapy has a role in the locally advanced disease remains a controversial area. A subsequent radiotherapy after neoadjuvant chemotherapy can be considered, even if the randomized phase 3 LAP07 trial showed no difference in terms of overall survival [82]. However, it could be hypothesized that patients whose disease does not spread while receiving chemotherapy might benefit from radiation therapy.

## 5. Novel Molecular Targets and Possible Implications for Treatment of Early-Stage Pancreatic Cancer

There is increasing evidence supporting the role of a tailored approach in the advanced setting based on molecular heterogeneity of PDAC and its influence on prognosis and treatment response [83,84]. Recently, germline BRCA mutations have been validated as a predictive factor of response to the PARP inhibitor olaparib after platinum-based chemotherapy in the metastatic setting [85]. Other therapeutic targets that are being investigated in advanced disease include homologous recombination repair deficiency (HRD), microsatellite instability, HER2/HER3, CDK4/6, NTRK fusions, KRAS G12C, and BRAF mutations [83,86,87].

To date, the decision algorithm in early-stage pancreatic cancer is essentially based on anatomical resection criteria and clinical patient features [88].

A genomic-driven approach could pave the way to the entrance of personalized medicine, even in the preoperative treatment of PDAC [89]. Indeed, the implementation of biomarkers at an earlier stage could help in identifying the best candidates to an upfront resection, and in helping clinicians in the decision making of the best neoadjuvant therapy.

To our knowledge, no prospective data are available; however, two retrospective studies have suggested an increase in pathological complete response rates and overall survival in germline BRCA mutated PDAC patients treated with platinum-based therapies [90,91].

Some trials including a biomarker selection in the neoadjuvant setting are ongoing. The PRIMUS-002 is a phase II study investigating two platinum containing regimens in resectable and borderline resectable PDAC with HRD signature [92]. A phase II feasibility study aims to determine possible biomarkers of the MAPK inhibitor cobimetinib and the PARP inhibitor olaparib also in a preoperative setting [93].

Preclinical evidence has proposed a potential synergy between radiotherapy and PARP inhibition, and further clinical studies are being investigated [94]. Huge efforts are being made in different countries in order to enhance the role of precision medicine in PDAC by the use of platforms (e.g., PRECISION-Panc in the UK, EPPIC in Canada, Precision Promise in the USA). The PIONEER-Panc study is a phase 2 study with a Bayesian platform design that will investigate novel therapeutic approaches in three clinical stage groups of early-stage pancreatic cancer [95].

Shifting the paradigm in localized pancreatic cancer from a “all-comer” to a personalized approach, based on one’s own molecular profile, could be even more important in a potentially curable setting, since it could reduce the mortality of this highly lethal disease.

## 6. Imaging Assessment of Response to Therapy: Where Are We Headed?

Surgical eligibility is reevaluated after neoadjuvant chemoradiotherapy, and the decision of surgery is determined by a multidisciplinary team discussion. Imaging follow-up with contrast-enhanced CT after neoadjuvant therapy is recommended to provide adequate staging and assessment of resectability status [6]. Radiological assessment after neoadjuvant chemoradiotherapy may be challenging due to the difficulty in the differentiation of normal post-treatment changes from residual tumor [96].

Validated radiological assessment criteria (e.g., RECIST) seem not suitable for PDAC, due to changes in peritumoral fat changes related to therapy, along with perivascular/perineural patterns of growth of the tumor. In many patients treated with neoadjuvant chemoradiotherapy, CT performed after treatment shows an increase in peripancreatic edema and/or fibrotic strands compared with pretreatment scans, which are difficult to be differentiated from the viable tumor itself and may cause overestimation of tumor peripancreatic fat infiltration [97]. Serial tumor size change proved to be insufficient for reliable response evaluation after neoadjuvant therapy for pancreatic tumor [98,99].

Neoadjuvant therapy reduces the accuracy of tumor restaging, but this effect seems not to affect the determination of resectability [89]. Nowadays, surgical resectability of PDAC after chemoradiotherapy is usually based on NCCN guidelines [6]. After neoadjuvant chemoradiotherapy, up to one third of patients with borderline resectable tumor may show regression to resectable disease, and upto 20% of patients with locally advanced tumor may show regression to resectable or borderline resectable disease [100]. Post neoadjuvant therapy resectability status is an independent predictor of R0 resection [100]. However, radiologic downstaging of resectability according to NCCN criteria may underestimate the achievement of R0 margins at surgery [101].

Therefore, many groups looked for imaging predictors of resectability and outcome, with different approaches and results. Tumor density variation before and after therapy does not seem to be useful for evaluating pancreatic tumor response [99,102]. Cassinotto et al. [102] showed that partial regression of tumor-vessel contact indicates suitability for surgical exploration, irrespective of the degree of decrease in tumor size or the degree of residual vascular involvement. Jeon et al. [100] demonstrated that a post-chemoradiotherapy tumor size equal or smaller than 2 cm and decrease in tumor-venous contact are independently associated with R0 resection. The result by Beleù et al. [103] showed that a 25 mm cut-off for tumor size corresponded to a 64% sensitivity, 78% specificity, and 69% accuracy in assessing R0 resection, and that each 5 mm increment of tumor major axis dimension corresponded to an odds ratio of 1.79 for R+ resection. Based on the above considerations, it has been suggested that a decrease in tumor size or vascular contacts, even partial or moderate, should prompt surgical exploration even in the case of initially locally advanced disease [96].

More recently, quantitative assessment and functional imaging have been investigated for assessing tumor response after chemo- or radiotherapy in this setting. The adoption of 18-FDG PET and PERCIST criteria has been investigated with mixed results [104,105,106]. Recently, Zimmermann et al. [107] and Yokose et al. [108] showed that the post- chemoradiotherapy SUVmax can be an effective predictor of prognosis and treatment response to neoadjuvant therapy for PDAC. PERCIST criteria proved to be more accurate than RECIST criteria for restaging pancreatic tumor after neoadjuvant therapy [108]. Preliminary studies have shown that some perfusion CT parameters (e.g., blood flow and permeability) may be a good indicator of histopathological response to chemoradiotherapy to PDAC [109,110]. In a prospective study by Hamdy et coll. [109], patients who were deemed responders to neoadjuvant chemotherapy and radiation therapy had significantly higher baseline blood flow than those who did not, thus suggesting that perfusion CT performed before chemo-radiation therapy can help predict the histopathologic response to therapy. Preliminary results also showed that dual energy perfusion CT might be helpful for preoperative assessment of PDAC with the possibility of tumor grade prediction [111], as well as for detection of recurrent PDAC, with recurrent tissue showing a tendency to lower blood-flow values [112]. Radiomics is nowadays being widely used in the oncologic research setting to derive quantitative biomarkers for diagnosis and tumor response assessment. In the setting of post-chemoradiotherapy for PDAC, some recent studies showed the potential role of textural features extracted from baseline pancreatic phase CT imaging of patients with potentially resectable pancreatic ductal adenocarcinoma and longitudinal changes in tumor heterogeneity as biomarkers for predicting histologic response to neoadjuvant therapy, and patient’s outcome, including resectability, prognosis, and disease-free survival [113,114,115,116,117]. Despite the very promising results of these radiomics studies, further studies in larger populations with validation datasets are still required before radiomics may be implemented in clinical practice.

## 7. Conclusions

To summarize, management of patients with pancreatic ductal adenocarcinoma involves a multidisciplinary team. A comprehensive state-of-the-art knowledge of clinical and imaging criteria for tumor resectability, predicting the risk of margin-positive resection, and tumor response after neoadjuvant therapy, as well as knowledge of current and future perspective in therapeutical management is essential for adiologists to have an effective dialogue with other physicians in the multidisciplinary board for the best care of patients with pancreatic cancer.

## Figures and Tables

**Table 1:** Main imaging findings of PDAC on ultrasound, CT, and MRI.

Imaging Technique	Imaging Findings
Ultrasound	‑ Tumor in pancreatic head: hypoechoic mass + double duct sign (dilatation of the pancreatic duct and dilatation of the bile duct) ‑ Tumor in body/tail: very difficult to be detected; if visible hypoechoic mass with upstream dilatation of the pancreatic duct ‑ Poor vascularity on Doppler-US
Computed Tomography	‑ Ill-defined hypoattenuating mass, abrupt ductal cut off at the site of the mass double duct sign, poor enhancement on pancreatic and venous phases compared to normal pancreatic parenchyma, tendency to isoattenuation to normal pancreatic parenchyma in delayed phases ‑ Isoattenuating mass in 5.4–11% of cases, mainly in case of small lesions, abrupt ductal cut off at the site of the mass
Magnetic Resonance Imaging	‑ Hypointense compared to normal pancreatic parenchyma on T1-weighted precontrast images, variable intensity on T2-weighted images, slower enhancement than the normal pancreas thus being hypovascular compared to normal pancreatic parenchyma on pancreatic and portal venous phases, and isovascular to normal pancreatic parenchyma in delayed phases, usually restricted diffusion on diffusion weighted images, abrupt ductal cut off at the site of the mass, double duct sign
